# An Unrecognized Bifid Median Nerve in Revision Carpal Tunnel Release: Lessons for Surgical Exploration

**DOI:** 10.1155/cro/9523047

**Published:** 2026-07-03

**Authors:** Daniel Lynch, Tyler Small, Dyllan Epstein, Usmaan Zunnu Rain, Zakariah Mahmood

**Affiliations:** ^1^ Department of Orthopaedic Surgery, University of Central Florida/HCA Ocala GME Consortium, Ocala, Florida, USA; ^2^ Department of General Surgery, University of Central Florida/HCA Ocala GME Consortium, Ocala, Florida, USA; ^3^ Department of Medical Education, Central Michigan University College of Medicine, Saginaw, Michigan, USA, cmich.edu

**Keywords:** bifid median nerve, carpal tunnel release, carpal tunnel syndrome, peripheral nerve, revision

## Abstract

When patients experience persistent or worsening symptoms within the first 6–12 weeks following carpal tunnel release (CTR), surgeons should remain vigilant for underlying anatomical variations such as a bifid median nerve (BMN), which may contribute to incomplete decompression if unrecognized. Postoperative imaging may fail to identify this variant, especially when bifurcation occurs proximally in the forearm. Surgeons should consider extended‐field magnetic resonance imaging (MRI) or high‐resolution ultrasound (US) in revision cases. Patients should be counseled on an extended surgical approach for adequate nerve decompression and identification of a potential variety of anatomical variants leading to these symptoms. We present a case of progressive postoperative symptom worsening after primary open CTR in which a proximally bifurcated BMN, missed on MRI, was identified during revision surgery.

## 1. Introduction

Carpal tunnel syndrome (CTS) is the most common compressive peripheral neuropathy, with an incidence of 0.99–3.46 cases per 100,000 people [1]. Nearly 500,000 carpal tunnel releases (CTR) are performed annually, with 1%–5% requiring revision for persistent or recurrent symptoms [2]. Pain typically improves first after CTR, followed by gradual sensory recovery over the first postoperative year. Persistent or worsening symptoms within 6–12 weeks of surgery should serve as a red flag for incomplete release or early recurrence. Excess scar formation causing nerve tethering or impaired gliding, as well as iatrogenic nerve injury, may contribute to these cases [3]. Patient factors such as tobacco use, diabetes, psychiatric conditions, and prior cubital tunnel release also increase recurrence risk [4]. Documented anatomic variants that predispose to incomplete decompression include a persistent median artery (PMA), accessory palmaris longus, and aberrant flexor digitorum superficialis slip [3].

A less common but clinically significant anatomical variant is the BMN, with a reported prevalence of 9%–26% [5]. Lanz classified median nerve variations by division and accessory branching patterns [6]. In the Type III BMN, bifurcation occurs in the distal forearm before entering the carpal tunnel, where the two bundles may occupy more space and heighten compression risk [7]. A BMN may predispose patients to CTS and may contribute to persistent symptoms following primary release. When present, it may coexist with a PMA, increasing the risk of iatrogenic injury or incomplete decompression of both bundles [7]. Routine use of preoperative ultrasound (US) or MRI, particularly in revision cases, has been recommended to detect such anomalies [8].

In this report, we present a patient with a BMN who experienced worsening carpal tunnel symptoms within 6 months of primary release. The variant was not identified during the initial surgery and was discovered intraoperatively during revision CTR. This case report was conducted in accordance with institutional guidelines, and informed consent was obtained from the patient for surgical treatment and publication of deidentified clinical data and images.

## 2. Case Report

An 80‐year‐old right‐hand–dominant male with a history of cervical radiculopathy (status‐post cervical decompression and fusion) was referred after undergoing open left CTR in early 2025. He reported worsening hand pain, numbness, and weakness, with complete sensory loss in the radial three digits of his left hand and decreased function during golf and daily activities. Examination revealed a positive Tinel′s and Phalen′s at the carpal tunnel and hypoesthesia in the median distribution. Finger flexion, extension, and thumb opposition were intact. Incisions were well‐healed without infection, though he had A1 pulley pain and triggering of the left middle finger.

Preoperative MRI was independently reviewed by both the treating hand surgeon and the patient′s prior neurosurgeon with attention to the carpal tunnel area. No bifurcation or anomalous median nerve anatomy was identified on initial review. In addition, the radiology report did not mention anomalous median nerve anatomy or median nerve injury. The patient′s MRI demonstrated thenar atrophy, edema, and fatty replacement (Figure 1) without identified anatomic abnormalities. Review by both surgeons confirmed residual scar tissue but no median nerve injury. Electromyography showed severe left CTS with underlying polyneuropathy and absent median nerve responses at the wrist and digits, as well as left C5–6 radiculopathy.

**Figure 1:**
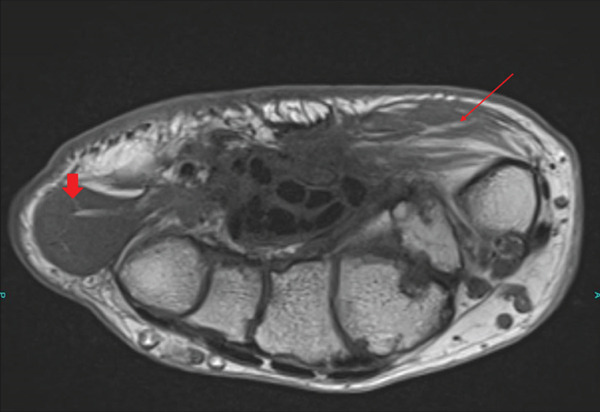
Preoperative T‐1 weighted MRI of the left hand showing atrophy and fatty streaking throughout the thenar musculature (arrow), indicating chronic denervation. Patient′s hypothenar musculature (arrowhead) shows robust muscle belly without degenerative changes.

Given persistent symptoms, the patient elected to undergo revision CTR. Possible external neurolysis, nerve repair, nerve wrapping, and hypothenar fat pad flap were discussed. Five months after the primary CTR, he underwent revision CTR with wound exploration, possible median nerve repair, and neurolysis, along with left middle finger A1 pulley release.

Intraoperatively, the A1 pulley was released via a 1‐cm diagonal incision over the volar middle finger and closed with nylon suture. The prior carpal tunnel incision was reopened and extended proximally. Dense scar tissue was encountered, confirming prior release of the transverse carpal ligament. Dissection was carried proximally beyond the carpal tunnel into the distal forearm to allow full visualization of the median nerve course. The median nerve appeared pale with perineural scarring. The ulnar nerve and artery were identified (Figure 2), and a separate nerve‐like structure was traced proximally, revealing a BMN (Figure 3). Circumferential external neurolysis was performed on both branches under loupe magnification to ensure complete decompression along their proximal and distal courses. Both branches were then wrapped with an Axogen nerve protector (Axogen, Alachua, Florida). After copious irrigation and hemostasis, the wound was closed, and a volar splint applied.

**Figure 2:**
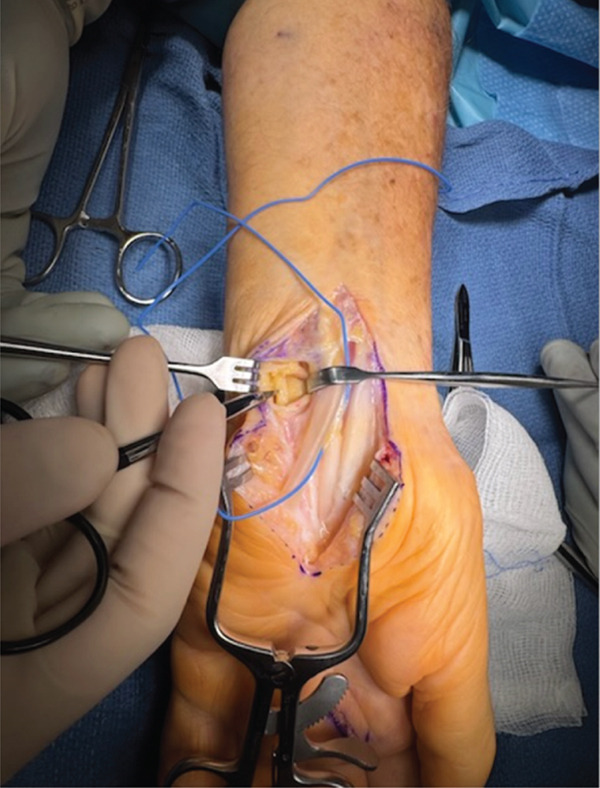
Intraoperative identification of the ulnar nerve (tips of tenotomy scissors). Due to the bifid nature of the median nerve, exploration of Guyon′s canal was performed to confirm that one of the bundles of the bifid median nerve was not an aberrant ulnar nerve in the forearm.

**Figure 3:**
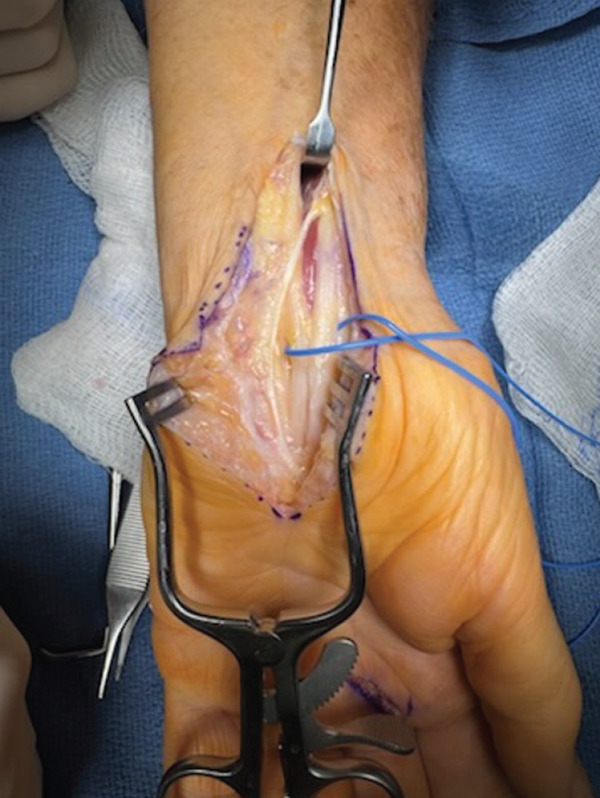
Intraoperative identification and tagging of a proximal bifid median nerve using a single vessel loop. A white, pale appearance to the nerve was noted along its course.

Postoperative outcomes were assessed clinically at routine follow‐up visits, focusing on sensory examination in the median nerve distribution, motor function and strength, patient‐reported functional improvement, and wound healing. At 2 weeks, sensation was unchanged, but hand function had improved; incisions were healed (Figure 4). At 8 weeks, he had stable median nerve symptoms with persistent hypoesthesia but continued functional improvement and full wrist and finger motion. Ongoing observation was recommended.

**Figure 4:**
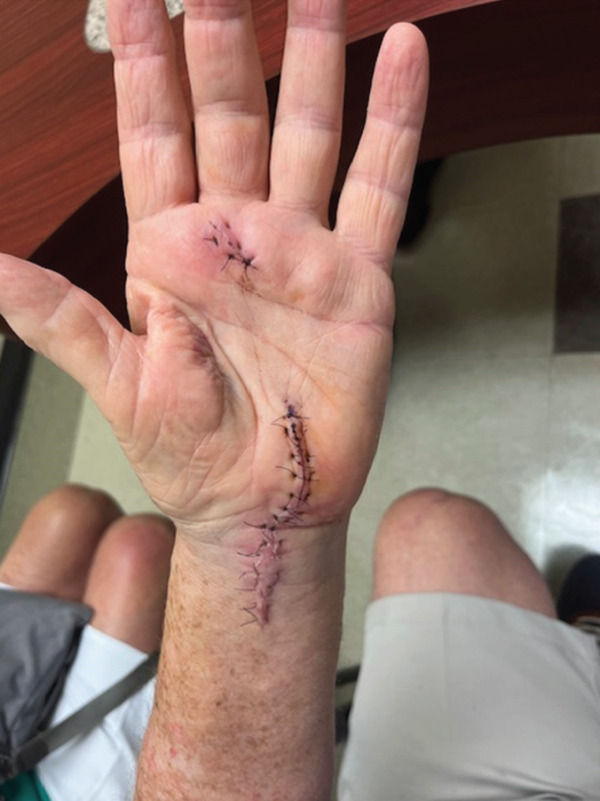
Two‐week postoperative clinical picture. Trigger finger release and extended CTR incisions are clean, dry, and intact without signs of infection.

## 3. Discussion

CTS is the most common compressive neuropathy of the upper extremity, and surgical decompression is generally effective. Many patients experience relief within days to weeks, particularly of nocturnal symptoms. Failure of expected postoperative symptom improvement after CTR occurs in up to 12% of patients and may indicate incomplete release or iatrogenic median nerve injury [9]. These cases warrant close follow‐up to determine whether observation or revision surgery is appropriate.

Anatomical variants are well‐recognized causes of incomplete decompression when not identified during the index procedure. Our case involved a proximal BMN that was not recognized on MRI or during the primary CTR, stressing the importance for an awareness of potential anatomical variants in revision cases. However, it has been shown that a BMN can be associated with cubital tunnel syndrome as well [10]. Other potential contributors include a PMA, accessory palmaris profundus, trifid median nerve, and aberrant flexor digitorum superficialis slip [11–14]. There are reports of the coexistence of a BMN, anomalous muscles, and a PMA contributing to CTS [15]. A proximal BMN can also cause forearm‐level compression (double‐crush phenomenon), further worsening symptoms. Once both fascicles of the BMN were identified and decompressed intraoperatively, our patient experienced functional improvement, though sensory recovery remained incomplete due to chronic compression.

A BMN is a relatively common structural variation of the median nerve but can be overlooked during revision planning. Preoperative imaging such as high‐resolution US or MRI may be beneficial in such cases. For our patient, the anomaly was not identified preoperatively, likely obscured by scar tissue. This emphasizes the value of surgeon‐directed imaging review rather than reliance on radiology reports alone. Early postoperative symptom recurrence (within 6–12 weeks) or acute worsening, as in our case, should prompt consideration of unrecognized anatomical variation or iatrogenic nerve injury.

Unlike prior reports where BMN led to persistent symptoms after CTR, our case illustrates acute *worsening* after the initial decompression [16, 17]. Corte et al. described the Lanz type IIIA BMN variant, where bifurcation occurs proximal to the carpal tunnel, causing incomplete decompression by a standard CTR [18]. Our findings support the consideration of such variants when evaluating failed CTR and to carefully assess preoperative imaging in revision cases.

Both US and MRI have value in the revision CTR setting. In a situation such as our case presentation, accurate and appropriate preoperative imaging can help avoid aggressive salvage surgeries with an appropriate preoperative plan [19]. US provides dynamic evaluation and low cost, whereas MRI, particularly MR neurography, offers superior soft‐tissue resolution for detecting scarring, neuroma, or deep structural abnormalities [20]. MRI may be preferred when complex pathology or space‐occupying lesions are suspected, though it is more expensive and less accessible. Studies show both modalities have comparable sensitivity for detecting recurrent compression, and combined use can be advantageous [21, 22]. The key remains a high index of suspicion for anatomical anomalies such as the proximal BMN identified in our case. One could argue that the utility of US to dynamically assess the integrity of the median nerve along its path more proximally may have allowed a better opportunity to identify the BMN prior to the revision surgery.

In patients with persistent or worsening symptoms after primary CTR, preoperative US or extended‐field MRI should be strongly considered. Surgeons should remain alert to anomalous nerve anatomy or iatrogenic injury and personally review all imaging. Retrospectively, the bifid nature of our patient′s nerve was identifiable on the MRI (Figure 5) only after intraoperative confirmation. However, it had been missed by the reading radiologist and two surgeons. This highlights the importance of high‐quality MR imaging studies as well as assuring the full course of the median nerve as it traverses the carpal tunnel is included. Patients should also be counseled about the possibility of a longer incision during revision surgery to permit complete exposure and decompression.

**Figure 5:**
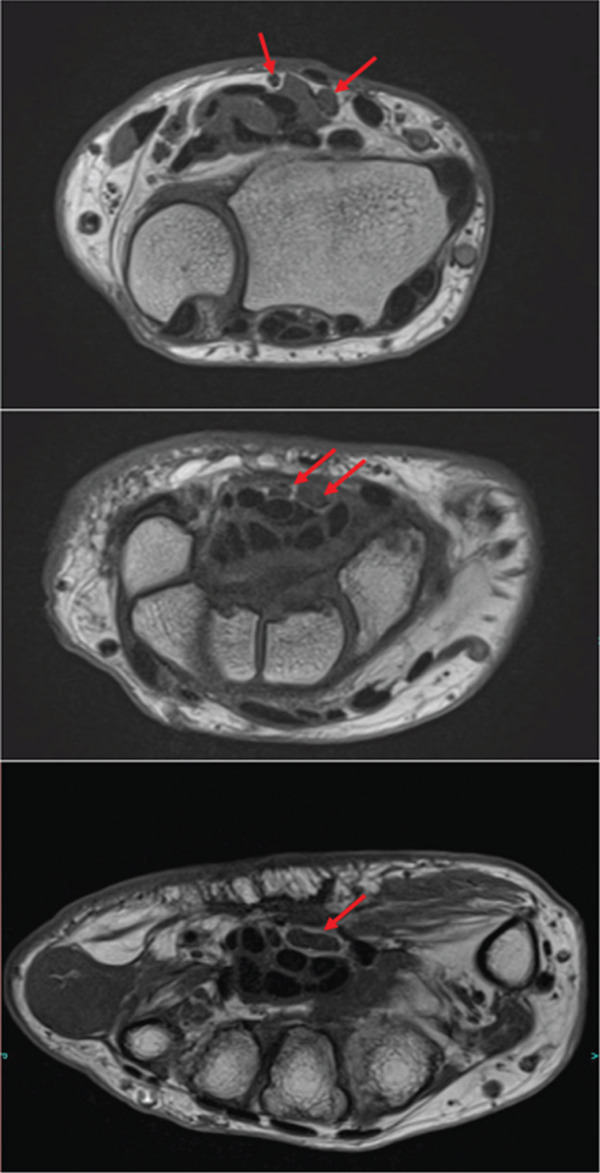
Preoperative T‐1 weighted MRI of the hand and wrist. Moving proximally (top) to distally (bottom), we observed the loss of the distinct bifid bundles of the median nerve (arrows) with confluence at the carpal tunnel (bottom), indicative of residual compression after the initial CTR.

This case highlights that structural variation of the median nerve, particularly a proximal BMN, should be considered in patients with early recurrence or worsening symptoms after CTR. In retrospect, it is possible that the median nerve tract that was not identified during the index surgery could have been mistaken for a superficial flexor tendon or a branch of the BMN that was damaged. Although most revision cases involve persistent symptoms, our patient experienced acute postoperative deterioration, a red flag for incomplete release or iatrogenic injury. Recognizing and addressing such variants intraoperatively may be important for optimal outcomes. Preoperative imaging, extended exposure, and surgical vigilance remain key principles in revision CTR.

Although we are unable to conclude that the BMN was directly correlated to the patient′s need for revision surgery, it is likely that it contributed to his symptoms due to the mass effect of two large nerve branches in a space (carpal tunnel) usually accommodating one median nerve branch. This contrasts with a prior report where the ulnar branch of a BMN was directly compressed by a palmaris profundus muscle, and resection of this anomalous muscle led to symptomatic improvement [23]. Intraoperatively, we were unable to identify a specific structure compressing the BMN. Along the course of the BMN, we did not observe any obvious flattening, caliber change, or focal compression of the BMN. However, residual fibrous tissue was readily prevalent at the site of the original CTR. Scar‐mediated tethering, ischemia, or iatrogenic injury may have contributed to the patient′s symptoms after the index procedure. Due to the index procedure only involving the standard CTR approach, we strongly believe incomplete proximal decompression was a contributing factor to the patient′s clinical course.

This report is limited by its single‐patient design, which precludes causal inference or generalization regarding the prevalence or clinical impact of a BMN in failed CTR. Additionally, postoperative outcomes were assessed clinically without validated patient‐reported outcome measures. Despite these limitations, this case highlights an important anatomical variant that may be overlooked and highlights the need for heightened suspicion in patients with early postoperative symptom worsening after CTR. The patient had underlying cervical radiculopathy as well as polyneuropathy. We understand that these confounders may have contributed to the patient′s baseline symptoms prior to CTR. However, due to the lack of subsequent treatment or surgical intervention for these pathologies in relation to his index and revision CTR, it can be assumed that his improvement after revision CTR was at least in part due to the further decompression of his BMN.

Prior to revision surgery, MRI and/or US should be utilized for preoperative planning to identify the potential cause. In revision cases, extended exploration and careful surgical planning are critical. We hope our case demonstrates the importance of extended exploration and awareness of anatomical variants during revision CTR, because although we discuss theoretically how a BMN can lead to persistent/worsening symptoms after CTR, we were unable to directly correlate his BMN to his clinical course. Unlike other reports of BMN identified preoperatively, our case involved an intraoperative diagnosis. Once identified, both bundles of the BMN were fully decompressed along their courses and wrapped in a nerve protector. Such anomalies may be missed even with US or MRI, underscoring the need for surgical vigilance and comprehensive preoperative planning.

## Funding

No funding was received for this manuscript.

## Conflicts of Interest

The authors declare no conflicts of interest.

## Data Availability

Data sharing is not applicable to this article as no datasets were generated or analyzed during the current study.

## References

[1] Cranford C. S. , Ho J. Y. , Kalainov D. M. , and Hartigan B. J. , Carpal Tunnel Syndrome, Journal of the American Academy of Orthopaedic Surgeons. (2007) 15, no. 9, 537–548, 10.5435/00124635-200709000-00004. 17761610

[2] Wessel L. E. , Gu A. , Asadourian P. A. , Stepan J. G. , Fufa D. T. , and Osei D. A. , The Epidemiology of Carpal Tunnel Revision Over a 1-Year Follow-Up Period, Journal of Hand Surgery. (2021) 46, no. 9, 758–764, 10.1016/j.jhsa.2021.04.003, 34059387. 34059387

[3] Westenberg R. F. , Oflazoglu K. , De Planque C. A. , Jupiter J. B. , Eberlin K. R. , and Chen N. C. , Revision Carpal Tunnel Release: Risk Factors and Rate of Secondary Surgery, Plastic and Reconstructive Surgery. (2020) 145, no. 5, 1204–1214, 10.1097/PRS.0000000000006742, 32332540. 32332540

[4] Nordstrom D. L. , DeStefano F. , Vierkant R. A. , and Layde P. M. , Incidence of Diagnosed Carpal Tunnel Syndrome in a General Population, Epidemiology. (1998) 9, no. 3, 342–345, 10.1097/00001648-199805000-00021, 9583428. 9583428

[5] Granata G. , Caliandro P. , Pazzaglia C. , Minciotti I. , Russo G. , Martinoli C. , and Padua L. , Prevalence of Bifid Median Nerve at Wrist Assessed Through Ultrasound, Neurological Sciences. (2011) 32, no. 4, 615–618, 10.1007/s10072-011-0582-8, 21533564. 21533564

[6] Lanz U. , Anatomical Variations of the Median Nerve in the Carpal Tunnel, Journal of Hand Surgery. (1977) 2, no. 1, 44–53, 10.1016/S0363-5023(77)80009-9. 839054

[7] Narayan S. , Bifid Median Nerve in a Patient With Carpal Tunnel Syndrome: A Case Report and Literature Review, Australasian Journal of Ultrasound in Medicine. (2016) 19, no. 4, 164–168, 10.1002/ajum.12029, 34760463. 34760463 PMC8409543

[8] Bayrak I. K. , Bayrak A. O. , Kale M. , Turker H. , and Diren B. , Bifid Median Nerve in Patients With Carpal Tunnel Syndrome, Journal of Ultrasound in Medicine. (2008) 27, no. 8, 1129–1136, 10.7863/jum.2008.27.8.1129. 18645070

[9] Gmainer D. G. , Hecker A. , Brinskelle P. , Draschl A. , Reinbacher P. , Kamolz L. P. , and Lumenta D. B. , Persistent Pain as an Early Indicator for Operative Carpal Tunnel Revision After Primary Release: A Retrospective Analysis of Recurrent and Persistent Carpal Tunnel Syndrome, Healthcare. (2023) 11, no. 14, 10.3390/healthcare11142100, 37510541. PMC10380003 37510541

[10] Asghar A. , Patra A. , Satish Ravi K. , Tubbs R. S. , Kumar A. , and Naaz S. , Bifid Median Nerve as an Anatomical Risk Factor for Carpal Tunnel Syndrome: A Meta-Analysis, Clinical Anatomy. (2022) 35, no. 7, 946–952, 10.1002/ca.23900, 35489054. 35489054

[11] Salter M. , Sinha N. R. , and Szmigielski W. , Thrombosed Persistent Median Artery Causing Carpal Tunnel Syndrome Associated With Bifurcated Median Nerve: A Case Report, Polish Journal of Radiology. (2011) 76, no. 2, 46–48, 22802832. 22802832 PMC3389914

[12] Kostoris F. , Bassini S. , Longo E. , and Murena L. , Carpal Tunnel Syndrome Associated With Bifid Median Nerve and Palmaris Profundus - Case Report and Literature Review, Journal of Hand Surgery. (2019) 24, no. 2, 238–242, 10.1142/S2424835519720123, 31035882. 31035882

[13] Oh S. , Trigger Wrist With Carpal Tunnel Syndrome Accompanied With Trifid Median Nerve: A Case Report and Literature Review, Archives of Plastic Surgery. (2022) 49, no. 6, 750–754, 10.1055/a-1947-2834. 36523910 PMC9747280

[14] Park C. H. , Lee Y. K. , and Koo K. H. , Lower Urinary Tract Infection and Periprosthetic Joint Infection After Elective Primary Total Hip Arthroplasty, Hip & Pelvis. (2017) 29, no. 1, 30–34, 10.5371/hp.2017.29.1.30, 28316960. 28316960 PMC5352723

[15] Qin J. , Tan X. X. , Xue M. Q. , Wang J. W. , Zhao J. M. , and Sha K. , Coexistence of Anomalous Muscle, Persistent Median Artery, Bifid Median Nerve Causing Carpal Tunnel Syndrome: A Case Report and Literature Review, Frontiers in Pediatrics. (2023) 11, 1043442, 10.3389/fped.2023.1043442, 36846165. 36846165 PMC9947498

[16] Depaoli R. , Coscia D. R. , and Alessandrino F. , In-Continuity Neuroma of the Median Nerve After Surgical Release for Carpal Tunnel Syndrome: Case Report, Journal of Ultrasound. (2015) 18, no. 1, 83–85, 10.1007/s40477-014-0127-0, 25767645. 25767645 PMC4353826

[17] Bhat A. K. , Acharya A. M. , Narayana Kurup J. K. , and Chakraborti A. , Bilateral Bifid Median Nerve With Bilateral Carpal Tunnel Syndrome: An Atypical Presentation, a Rare Cause and a Familiar Disease, Journal of Hand Surgery. (2018) 23, no. 2, 274–277, 10.1142/S2424835518720165. 29734910

[18] Corte E. L. , Gelmi C. A. E. , and Acciarri N. , Carpal Tunnel Syndrome Caused by the Entrapment of a Bifid Lanz IIIA Type Anatomical Variant of Median Nerve: A Case Report and Systematic Literature Review, Surgical Neurology International. (2021) 12, 10.25259/SNI_765_2020, 33598353. PMC7881513 33598353

[19] Kim Y. , Ko H. C. , and Son B. C. , Carpal Tunnel Syndrome Caused by High Division of the Median Nerve Proximal to the Carpal Tunnel: A Case Report, Nerve. (2018) 4, no. 1, 1–4, 10.21129/nerve.2018.4.1.1.

[20] Bagga B. , Sinha A. , Khandelwal N. , Modi M. , Ahuja C. K. , and Sharma R. , Comparison of Magnetic Resonance Imaging and Ultrasonography in Diagnosing and Grading Carpal Tunnel Syndrome: A Prospective Study, Current Problems in Diagnostic Radiology. (2020) 49, no. 2, 102–115, 10.1067/j.cpradiol.2019.04.004, 31126663. 31126663

[21] Tulipan J. E. , Kachooei A. R. , Shearin J. , Braun Y. , Wang M. L. , and Rivlin M. , Ultrasound Evaluation for Incomplete Carpal Tunnel Release, Hand. (2020) 15, no. 6, 780–784, 10.1177/1558944719832040, 30857423. 30857423 PMC7850243

[22] Megalaa B. S. , Ghany A. F. A. E. , Hetta W. M. , and Din N. M. H. E. , Role of Ultrasound and Magnetic Resonance Neurography in the Detection of Median Nerve Abnormalities in Carpal Tunnel Syndrome, Egyptian Journal of Radiology and Nuclear Medicine. (2024) 55, no. 1, 10.1186/s43055-024-01345-5.

[23] Yamazaki T. , Matsuura Y. , Takahashi H. , and Ohtori S. , A Rare Case of Carpal Tunnel Syndrome with Bifid Median Nerve and Palmaris Profundus Muscle, Plastic and Reconstructive Surgery–Global Open. (2024) 12, no. 10, e6228, 10.1097/GOX.0000000000006228, 39399799. 39399799 PMC11469878

